# Editorial: The link between nutrition and schizophrenia

**DOI:** 10.3389/fpsyt.2022.1074120

**Published:** 2022-11-21

**Authors:** Pek Yee Tang, Shiau Foon Tee, Kuan Pin Su

**Affiliations:** ^1^Department of Mechatronics and Biomedical Engineering, Lee Kong Chian Faculty of Engineering and Science, Universiti Tunku Abdul Rahman, Kajang, Malaysia; ^2^Department of Chemical Engineering, Lee Kong Chian Faculty of Engineering and Science, Universiti Tunku Abdul Rahman, Kajang, Malaysia; ^3^Departments of Psychiatry and Mind-Body Interface Laboratory (MBI-Lab), China Medical University Hospital, Taichung, Taiwan; ^4^An-Nan Hospital, China Medical University, Tainan, Taiwan; ^5^College of Medicine, China Medical University, Taichung, Taiwan

**Keywords:** schizophrenia, nutrition, diet, obesity, leptin, gut microbiome, orexins, antipsychotic

Schizophrenia is a complex disorder with many potential causes. The literature suggests a combination of genetic factors and environmental factors such as stress, diet, physical inactivity and drugs contribute to the development of schizophrenia. Recently, there have been reports that nutritional factors are important determinants of mental health (1, 2). It is known that the risk of developing psychosis could be affected by the composition of diets (3). Moreover, poor dietary practices in patients with schizophrenia (4–6) could be due to dysregulation of the reward circuitry in the mesolimbic pathway and cognition segments in the brain (7, 8). This Research Topic focuses on the relationship between nutrition and schizophrenia that will aid in the nutritional interventions for the amelioration and treatment of schizophrenia.

DNA methylation is one of the regulatory factors that control gene expression. DNA methylation of CpG islands leads to gene silencing, whereas hypomethylation of non-CpG island-associated promoters tends to deregulate gene expression (9, 10). The findings of Keleher et al. (11) reported that 4,356 genes were differentially expressed and methylated in response to a high-fat diet. One of these genes is *LEP* (or *OB*) which encodes leptin. This adipose-derived hormone is substantially involved in the regulation of energy balance and body weight (12). In this Research Topic, Song et al. reported significantly higher *LEP*-CpG7, *LEP*-CpG15 methylation in inpatients with schizophrenia. In addition, negative correlations were found between *LEP*-CpG7 methylation and PANSS positive subscore. Furthermore, *LEP*-mRNA expression was negatively correlated with PANSS total score and positive subscale. Their results indicated that leptin, which induces potential hippocampal synaptic neuroplasticity, could play a great role in the pathophysiology of schizophrenia.

An increasing body of evidence suggests that the gut microbiome has a profound impact on the development and progression of obesity. Dysbiosis, characterized by a lower diversity and alterations in the composition of the gut microbiome, is associated with overweight and obesity (13, 14). Current data suggest that people with severe mental illness have a markedly high prevalence of obesity than the general population (15). As such, Tsai et al. observed a significantly lower richness of gut microbiota at the class level in female schizophrenia patients with central obesity than in those with normal weight. These patients also showed lower alpha diversity at both phylum and class levels. At phylum level, the abundance of Verrucomicrobia was lower, while reduced abundance of *Akkermansia* and increased of *Prevotella* and *Roseburia* were observed at the genus level. Since antipsychotic medications were reported to contribute to the high prevalence of obesity in schizophrenia patients (16), their findings suggest the role of the gender factor in antipsychotic related gut dysbiosis. Meanwhile, Liang et al. addressed the alteration of microbial enterotypes in schizophrenia patients with a higher BMI. There were significant differences in enterotype-*Prevotella* (P) abundance between the obese schizophrenia patients and healthy controls. Proteobacteria and Firmicutes were significantly abundant in these patients with enterotype-P. Contrastingly, Bacteroidetes were highly abundant in health controls with enterotype-P. Their study revealed the enterotype-P might be crucial in a variety of metabolic pathways. The differences in enterotype-P abundance indicate disturbances of glucose and lipid metabolism in the obese schizophrenia patients.

Orexins are hypothalamic neuropeptides that are able to promote feeding (17), regulate sleep/wake cycles (18) and autonomic function, such as blood pressure and heart rate (19). Orexin cells are activated by fasting and low glucose levels, and drive eating until ingested glucose inactivates them (20, 21). Patients with schizophrenia had a higher orexin A level, but the causal linkage remains debatable (22, 23). Therefore, Li et al. conducted a meta-analysis on the alteration of plasma orexin-A levels in patients with schizophrenia. However, their meta-analysis did not show any significant alteration in plasma orexin-A levels between patients with schizophrenia and healthy controls. The non-significant results could be attributed to the small number of studies and sample size, high heterogeneity across studies and a lacked of detailed information.

Current evidence shows that schizophrenia patients are likely to be overweight or obese. The larger challenge remains how to determine the possible links between side effects of anti-psychotic drugs and poor dietary patterns, and metabolic syndrome in schizophrenia patients. Due to limited research, observational, mechanistic and interventional studies are essential to determine the precision of dietary manipulation in schizophrenia prevention and management.

## Author contributions

The editorial was drafted by PT and further edited by ST and KS. All authors contributed to the article and approved the submitted version.

## Conflict of interest

The authors declare that the research was conducted in the absence of any commercial or financial relationships that could be construed as a potential conflict of interest.

## Publisher's note

All claims expressed in this article are solely those of the authors and do not necessarily represent those of their affiliated organizations, or those of the publisher, the editors and the reviewers. Any product that may be evaluated in this article, or claim that may be made by its manufacturer, is not guaranteed or endorsed by the publisher.
